# Supplementary figures and images for: Maintenance of neuronal TDP-43 expression requires axonal lysosome transport
Source: eLife. 2025 Sep 19;14:RP104057. doi: 10.7554/eLife.104057 (PMC12448747; doi:10.7554/eLife.104057)

250 kDa  
150 kDa  
100 kDa  
50 kDa  
37 kDa  
25 kDa  
20 kDa  
15 kDa  
10 kDa

sg100

sg200

sg100

sg200

sg100

sg200

sg100

sg200

sg100

sg200

sg100

sg200

Halo-TDP-43

endogenous TDP-43

RPL24

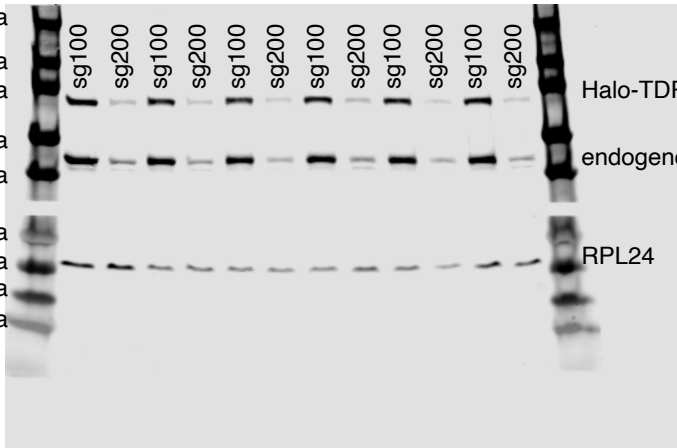

Supplement: Figure 1—source data 1. [file elife-104057-fig1-data1.zip › Image_Halo-TDP43kd.pdf]

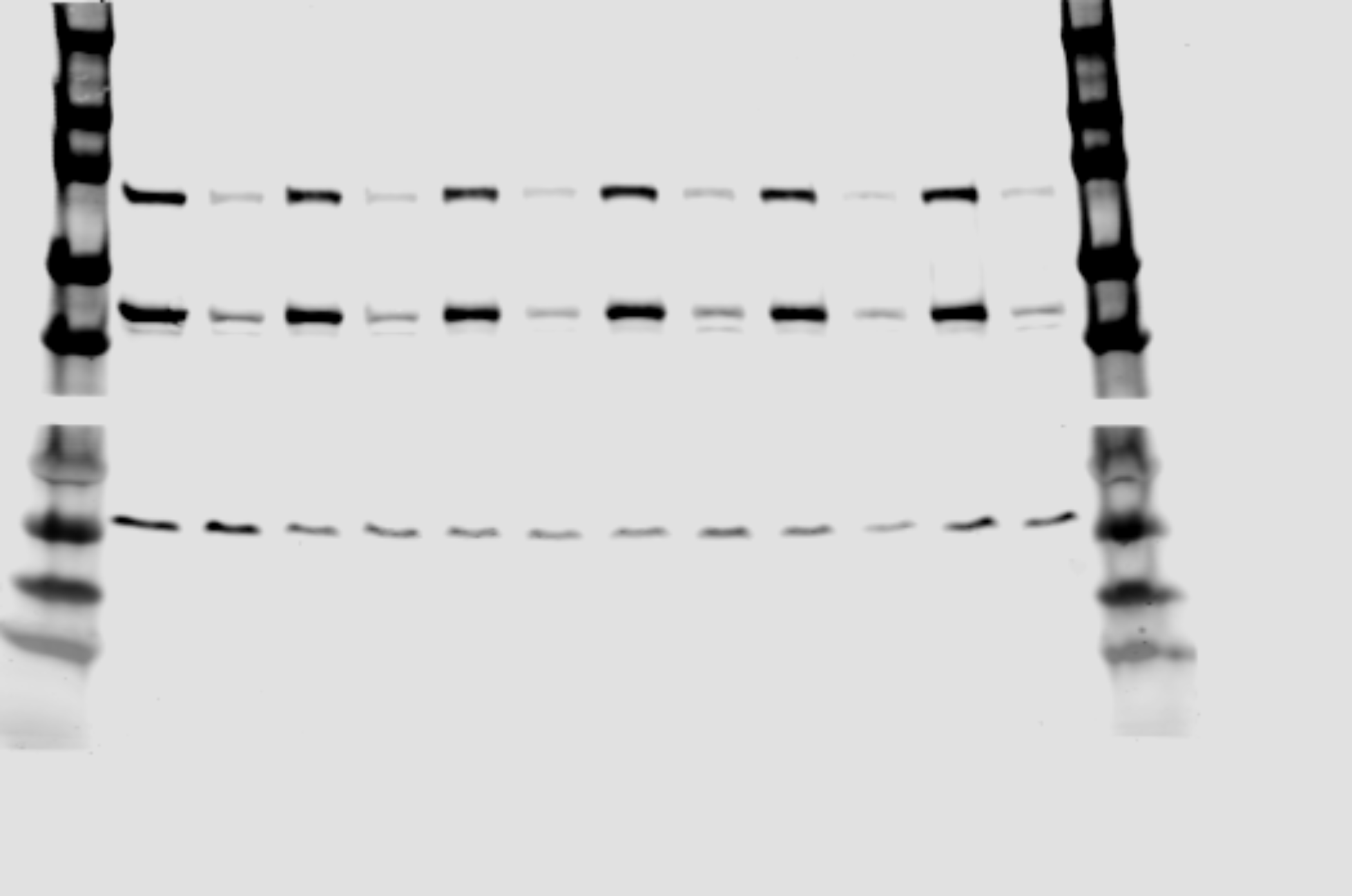

Supplement: Figure 1—source data 2. [file elife-104057-fig1-data2.zip › Image_Halo-TDP43kd.tif]

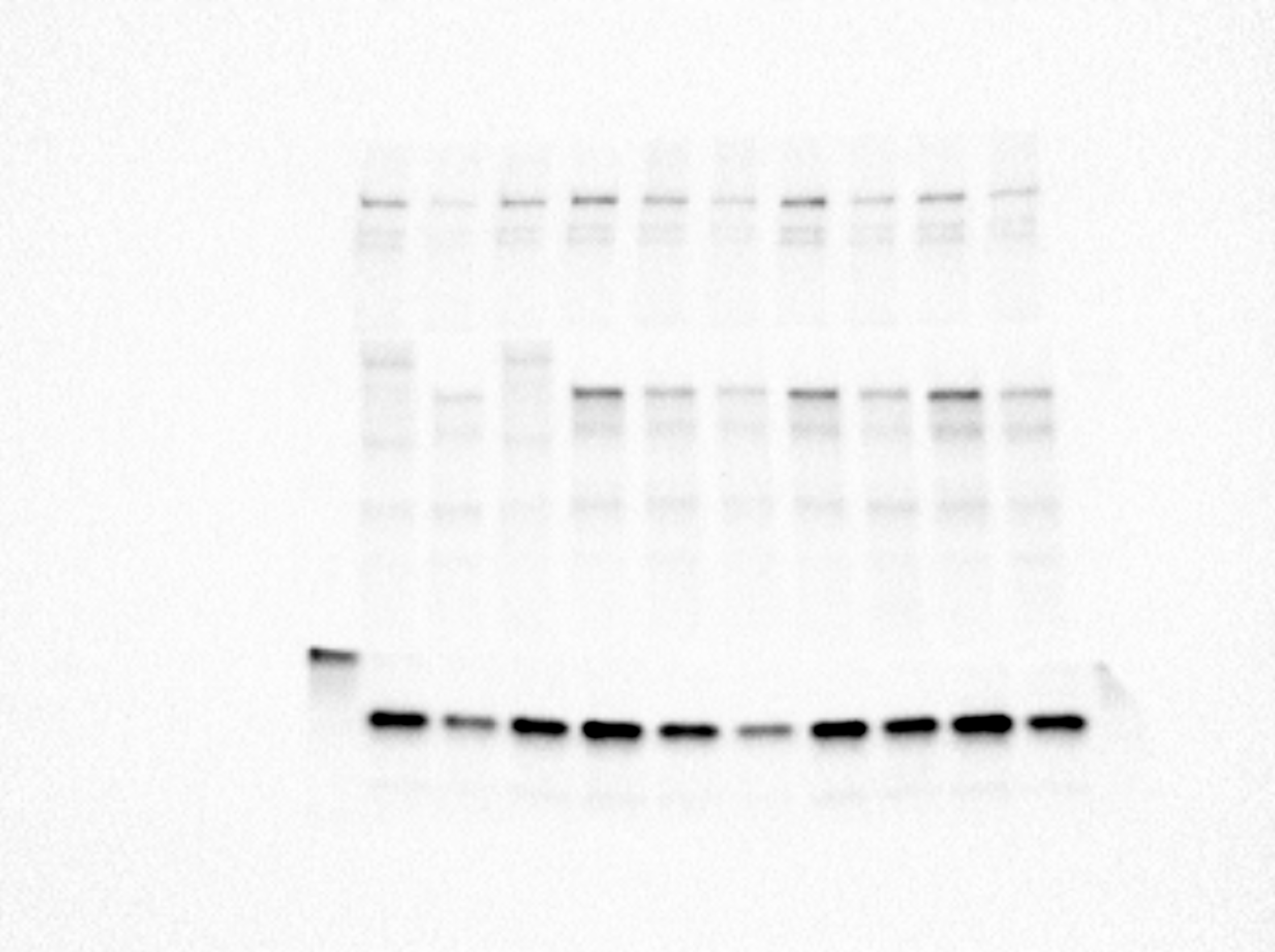

Supplement: Figure 1—figure supplement 1—source data 2. [file elife-104057-fig1-figsupp1-data2.zip › fernandopullems 2021-01-29_15h26m08s_Exposure_5.0sec.tif]

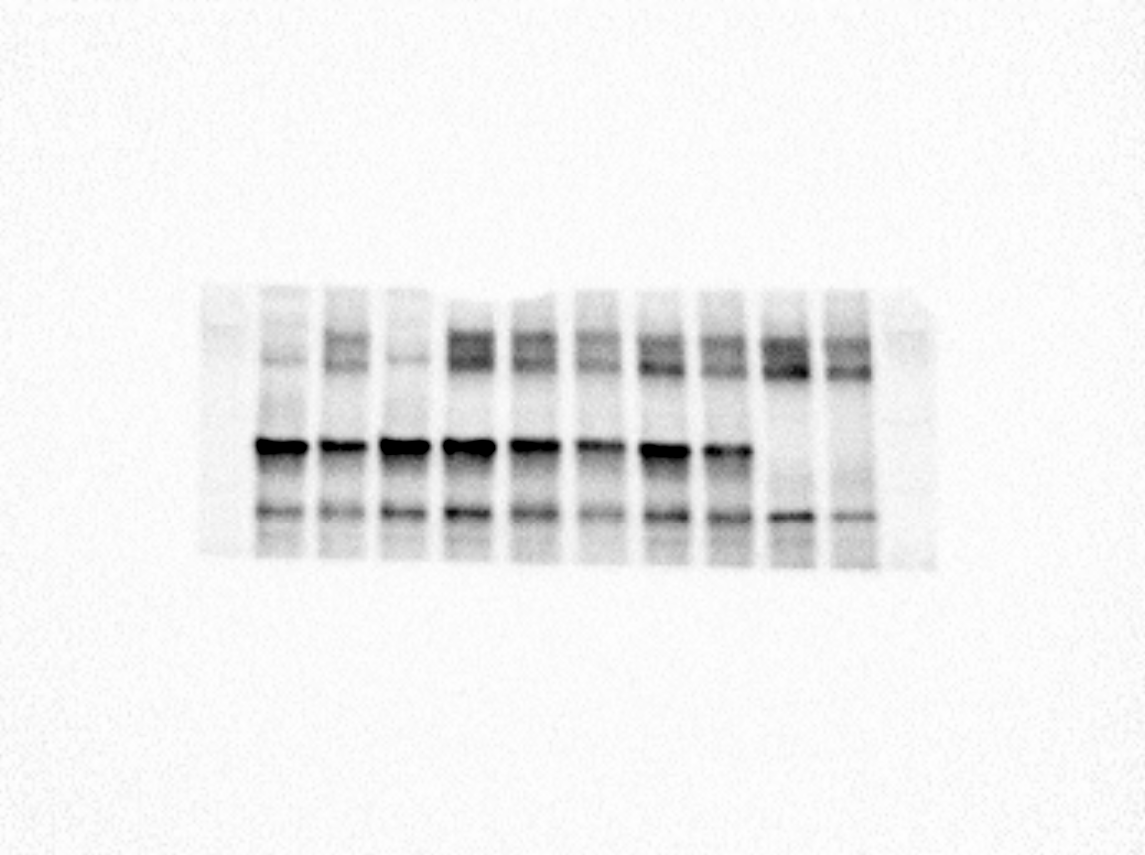

Supplement: Figure 1—figure supplement 1—source data 2. [file elife-104057-fig1-figsupp1-data2.zip › TDP43 reblot. 2021-02-09_14h30m08s_Exposure_1.2sec.tif]
