# Supplementary material for: Maintenance of neuronal TDP-43 expression requires axonal lysosome transport
Source: eLife. 2025 Sep 19;14:RP104057. doi: 10.7554/eLife.104057 (PMC12448747; doi:10.7554/eLife.104057)
Supplement: Figure 1—figure supplement 1—source data 1. [file Figure1Supplement_sourcedata1.pdf › Figure1Supplement_sourcedata1.pdf]

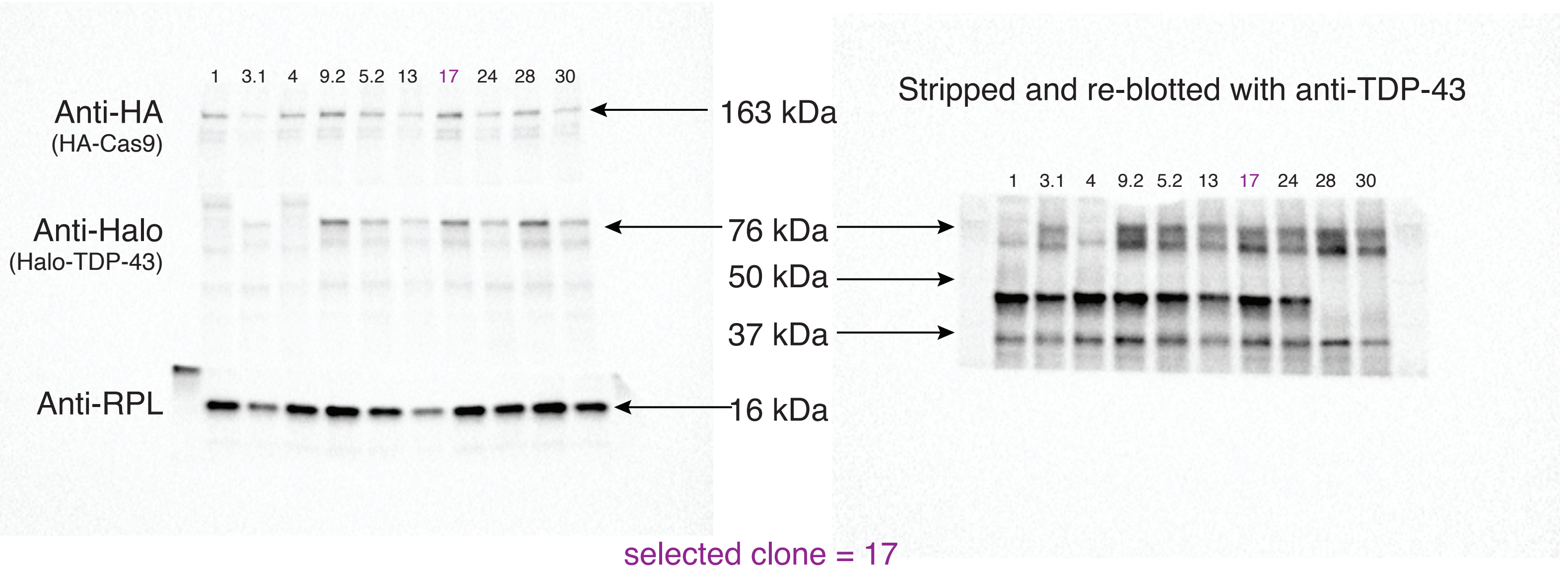
1
3.1
4
9.2
5.2
13
17
24
28
30
Anti-HA
(HA-Cas9)
163 kDa
Anti-Halo
(Halo-TDP-43)
76 kDa
50 kDa
37 kDa
Anti-RPL
16 kDa
Stripped and re-blotted with anti-TDP-43
1
3.1
4
9.2
5.2
13
17
24
28
30
selected clone = 17
